# Weary Nights

**Published:** 1890-05-24

**Authors:** 


### Words of Consolation.

### WEARY NIGHTS.

In our time of health, which perhaps has long gone by, we looked forward to the night with pleasure and longing, knowing that by it we should renew our strength, refresh our weariness, and for a season be free from our daily worries and vexations. But, alas! now everything is changed, the hours of darkness bring only fresh suffering, with a peculiar languor, restlessness, and sleeplessness. There is no falling into a delightful drowziness the instant our head is on the pillow—rather, we seem to get wider awake the more we court sleep, and as the hours go on we become the victims of all sorts of terrors and fears which are worse even than pain. The nerves are unstrung, all our fears for ourselves or others start up afresh, and with them our most unloving, saddest, murmuring, and discontented thoughts arise. Every prospect for this world and the next looks black and dismal. We shall never be well again, we complain. Such suffering as this must end in death, and then what hope of salvation is there for such a miserable sinner! Then tossing on our bed, we groan, "Would God that it were morning," though we know too well that after a restless and troubled sleep we shall be ready in the morning to cry, "Would God that it were evening!"

Alas! for those who can find no help, no solace, in prayer to God. Do you, dear friends, dread the coming of the night, and say, with Job, wearisome nights are appointed me? Think well on these complaining words, and light will come out of the darkness; for in reality they bring with them the truest comfort. Our sorrows are *appointed* for us. They do not come by chance or accident; God has ordered them for us. He is about our bed, and knows what we need and how much we can bear, and will not put on us more than is really necessary for our perfection. He watches over us, and makes all our bed in our sickness. That couch so uneasy and comfortless shall be smoothed by His loving hand. Those strange fancies and sights conjured up by our poor weak brains, which frighten us as though they were realities, shall pass away at the breath of His Spirit. No, there *is* no way of meeting our trials but by looking at these wearisome nights as appointed by God. Try, then, and realise that it is His will, and fly to Him for the comfort which He promises you. Your weariness and weakness cannot be greater than was our Blessed Lord's in the fearful night of agony in the garden. He prayed amidst His bitter anguish that the cup might pass away, but if not that His Father's will might be done. Do you then go in the same spirit to the Throne of Grace, and an angel from heaven in like manner will strengthen you.

